# Oral Microbiome Profiles: 16S rRNA Pyrosequencing and Microarray Assay Comparison

**DOI:** 10.1371/journal.pone.0022788

**Published:** 2011-07-29

**Authors:** Jiyoung Ahn, Liying Yang, Bruce J. Paster, Ian Ganly, Luc Morris, Zhiheng Pei, Richard B. Hayes

**Affiliations:** 1 Division of Epidemiology, Department of Environmental Medicine, New York University School of Medicine, New York, New York, United States of America; 2 Department of Pathology, New York University School of Medicine, New York, New York, United States of America; 3 Department of Molecular Genetics, The Forsyth Institute, Cambridge, Massachusetts, United States of America; 4 Harvard School of Dental Medicine, Boston, Massachusetts, United States of America; 5 Department of Head and Neck Surgery, Memorial Sloan-Kettering Cancer Center, New York, New York, United States of America; Columbia University, United States of America

## Abstract

**Objectives:**

The human oral microbiome is potentially related to diverse health conditions and high-throughput technology provides the possibility of surveying microbial community structure at high resolution. We compared two oral microbiome survey methods: broad-based microbiome identification by 16S rRNA gene sequencing and targeted characterization of microbes by custom DNA microarray.

**Methods:**

Oral wash samples were collected from 20 individuals at Memorial Sloan-Kettering Cancer Center. 16S rRNA gene survey was performed by 454 pyrosequencing of the V3–V5 region (450 bp). Targeted identification by DNA microarray was carried out with the Human Oral Microbe Identification Microarray (HOMIM). Correlations and relative abundance were compared at phylum and genus level, between 16S rRNA sequence read ratio and HOMIM hybridization intensity.

**Results:**

The major phyla, Firmicutes, Proteobacteria, Bacteroidetes, Actinobacteria, and Fusobacteria were identified with high correlation by the two methods (r = 0.70∼0.86). 16S rRNA gene pyrosequencing identified 77 genera and HOMIM identified 49, with 37 genera detected by both methods; more than 98% of classified bacteria were assigned in these 37 genera. Concordance by the two assays (presence/absence) and correlations were high for common genera (*Streptococcus, Veillonella, Leptotrichia, Prevotella, and Haemophilus*; Correlation = 0.70–0.84).

**Conclusion:**

Microbiome community profiles assessed by 16S rRNA pyrosequencing and HOMIM were highly correlated at the phylum level and, when comparing the more commonly detected taxa, also at the genus level. Both methods are currently suitable for high-throughput epidemiologic investigations relating identified and more common oral microbial taxa to disease risk; yet, pyrosequencing may provide a broader spectrum of taxa identification, a distinct sequence-read record, and greater detection sensitivity.

## Introduction

The NIH Human Microbiome Project, launched as part of the NIH Common Fund's Roadmap for Medical Research, pointed to the need to accelerate our understanding of how our bodies and microorganisms interact to influence health and disease [1]. The oral microbiome plays a critical role in the maintenance of a normal oral physiological environment and in development of oral diseases, including periodontal disease [2] and tooth loss [3]. Although little studied, the oral microbiome may be important in cancer and other chronic diseases, through direct metabolism of chemical carcinogens and through systemic inflammatory effects [4].

With the characterization of microbial genetic profiles, molecular technologies can elucidate microbial community structure, including the identification and quantification of culturable and non-culturable organisms, at a much higher resolution than was previously possible with culture-based methods. Complete genetic sequencing of complex microbial ecosystems in humans have been accomplished [5], however, higher-throughput methods are needed for larger-scale epidemiologic investigations relating microbiome profiles to disease risk. The major approaches to cost-efficient high-throughput characterization of the human microbiome exploit the high variability in microbial 16S ribosomal RNA (rRNA) gene sequence, uniquely found in prokaryotes and considered as a barcode that can be used to identify specific microbes, characterizing the broad spectrum of both culturable and non-culturable organisms. The development of these methods has opened the possibility of conducting large population-based studies of human microbiome, providing insight into the diversity and community structure of the human microbiome in relation to health and disease. Our interest is in the 16S rRNA gene pyrosequencing assay [6] and the Human Oral Microbe Identification Microarray (HOMIM) hybridization assay [7], two well-validated methods for microbiome profiling by assessment of microbial 16S rRNA gene diversity in human samples, with pyrosequencing selected as a broad-based approach applicable generally to the microbiome and HOMIM focused specifically on the oral microbiome.

16S rRNA gene pyrosequencing has been applied in a wide range of human microbiome studies. Briefly, DNA primers to highly conserved regions in the 16SrRNA gene are designed for PCR amplification of DNA product, followed by DNA sequencing for characterization of microbial communities, including non-identifiable types, based on DNA sequence in the highly variable inter-primer regions. We (LY and ZP) have designed and validated a 16S rRNA pyrosequencing assay for the V3–V5 region of the gene and reported that 347F/803R is the most suitable pair of primers for classification of the foregut microbiome [6].

The Human Oral Microbe Identification Microarray (HOMIM) is a custom array-based approach developed by one of us (BP) and others at the Forsyth Institute (Cambridge, MA), using specially designed probes to detect ∼300 of the most prevalent oral bacterial species, initially identified from Sanger sequencing (http://mim.forsyth.org/). The approach is based on 16S rRNA gene sequence hybridization and has been extensively validated [3], [8]. Since this method is based on a pre-constructed microarray, the community structure identified is for the specific hybridization probes selected for previously identified bacteria.

Here, we quantitatively compare the two oral microbiome survey methods: broad-based16S rRNA gene pyrosequencing and custom 16S rRNA hybridization (HOMIM) as methods for microbiome characterization suitable for epidemiologic investigations.

## Results

With 16S rRNA gene pyrosequencing, we recovered ∼79,000 sequences from the 20 oral wash samples (Table S1), with 11 bacterial phyla detected (Table 1), including Firmicutes, Proteobacteria, Bacteroidetes, Actinobacteria, and Fusobacteria as the major phyla accounting for 99.83% of the distribution. Although pyrosequencing additionally detected SR1, TM7, Cyanobacteria, Spirochaetes, Tenericutes, and Synergistetes, the sum of these comprise only 0.16% of the total sample.

**Table 1:** The relative abundance correlation of phyla: data from 16S rRNA gene pyrosequencing and Human Oral Microbe Identification Microarray (HOMIM) assay.

	Relative Distribution (%)*		Correlation
Phylum	Pyrosequencing	HOMIM	
Firmicutes	52.30	45.69	0.80
Proteobacteria	19.67	16.38	0.80
Bacteroidetes	15.63	18.10	0.86
Actinobacteria	7.27	10.34	0.70
Fusobacteria	4.96	6.03	0.76
SR1	0.07	0.00	ND**
TM7	0.06	0.86	ND
Cyanobacteria	0.01	0.00	ND
Spirochaetes	0.01	2.59	ND
Tenericutes	0.01	0.00	ND
Synergistetes	0.0001	0.00	ND
Total	100.0	100.0	

*Relative Distribution was calculated after exclusion of 3.2% unclassified bacteria.

**ND: non determined.

Note: P value for chi-square test of the relative abundance distribution = 0.65.

**Figure 1:**
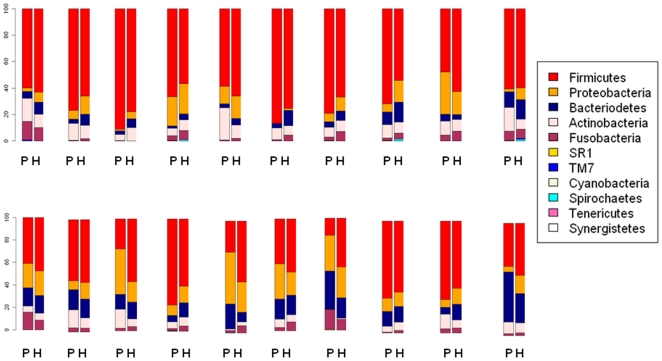
The relative abundance of phyla for each subject (n = 20). P indicates 16S rRNA gene pyrosequencing results and H indicates Human Oral Microbe Identification Microarray (HOMIM) assay results. Unclassified bacteria in 16S rRNA gene pyrosequencing are excluded.

HOMIM assay detected 7 phyla. As with pyrosequencing, Firmicutes, Proteobacteria, Bacteroidetes, Actinobacteria, and Fusobacteria were the major phyla identified by HOMIM, accounting for 96.5% of the distribution. No phyla were detected by HOMIM that were undetected in pyrosequencing, with the relative distribution being similar by the two methods (Chi square p value = 0.65). Correlations for abundance, comparing continuous level 16S rRNA sequence read ratio and HOMIM relative intensity, for the five major phyla ranged from r = 0.70 to r = 0.86. Relative abundance of phyla from pyrosequencing and HOMIM assay are shown for each individual in Figure 1.

The 16S rRNA gene pyrosequencing detected 77 genera (Table 2). In addition, 17.5% of the pyrosequencing reads were assigned by RDPII as unclassified taxa. HOMIM detected 49 genera, including 37 detected by both methods. As shown in Table 2 (and Table S2), >98% of classifiable bacteria in the subject samples were assigned in these 37 genera by both methods. Although 16S rRNA sequencing detected an additional 40 classifiable genera not identified by HOMIM microarray, most of these are found at very low densities (1.6% of identified bacteria), after exclusion of 17.5% unclassified taxa. Similarly, an additional 12 genera were detected only by HOMIM; their contribution to the overall percentage was also small (1.9%), yet this may represent false positives on the HOMIM due to cross hybridization on short prove reads. This could also be due to the misidentification or incorrect nomenclature from the RDP database.

**Table 2:** Number and relative distribution of known genera detected by 16S rRNA gene pyrosequencing and Human Oral Microbe Identification Microarray (HOMIM) assay.

	Pyrosequencing	HOMIM
Genera detected by both pyrosequencing and HOMIM	N = 37 (98.4%)*	N = 37 (98.1%)
Genera detected only by pyrosequencing	N = 40 (1.6%)*	NA
Genera detected only by HOMIM	NA	N = 12 (1.9%)

*Percentages of relative distribution were calculated after exclusion of 17.5% unclassified taxa from RDPII.

We compared concordance and correlations of genera detected by the both assay methods. *Streptococcus, Veillonella, and Leptotrichia*, were positive by pyrosequencing and HOMIM in most study subjects (20 positive for *Streptococcus and Veillonella*; 19 positive for *Leptotrichia*) with 100% concordance (Table 3) and high correlations for relative abundance (r = 0.71–0.80). Concordance of *Prevotella*, *Haemophilus*, *Capnocytophaga*, *Granulicatella*, *Lactococcus*, *Camplylobacter*, *Gemella*, *Neisseria*, *Fusobacterium*, *Parvimonas*, *Kingella*, and *Mycoplasma* were 85% or higher. For less common genera, including *Atopobium*, *Slakia*, and *Filifactor*, concordance and correlations were low or modest. As expected, relatively uncommon genera were more likely to be positive in pyrosequencing methods, but to be negative in HOMIM which was designed to identify the more common bacterial forms.

**Table 3:** Concordance and correlation of 37 genera detected from 16S rRNA gene pyrosequencing and Human Oral Microbe Identification Microarray (HOMIM) assay.

	% concordant rate^a^	Concordant		Discordant		Correlation^b^
		HOMIM positive	HOMIM Negative	HOMIM Negative	HOMIM Positive	
		PyrosequencingPositive	PyrosequencingNegative	PyrosequencingPositive	PyrosequencingNegative	
*Streptococcus*	100	20	0	0	0	0.80
*Veillonella*	100	20	0	0	0	0.71
*Leptotrichia*	100	19	1	0	0	0.77
*Prevotella*	95	19	0	1	0	0.84
*Haemophilus*	95	18	1	0	1	0.70
*Capnocytophaga*	95	16	3	0	1	0.29
*Rothia*	90	18	0	2	0	0.67
*Granulicatella*	90	17	1	2	0	0.31
*Lactococcus*	90	1	17	2	0	0.01
*Camplylobacter*	85	17	0	3	0	0.79
*Gemella*	85	17	0	1	2	0.62
*Neisseria*	85	16	1	1	2	0.69
*Fusobacterium*	85	15	2	3	0	0.72
*Parvimonas*	85	12	5	0	3	0.89
*Kingella*	85	6	11	2	1	0.52
*Mycoplasma*	85	1	16	3	0	0.97
*Bifidobacterium*	80	4	12	1	3	0.52
*Scardovia*	80	1	15	3	1	0.75
*Catonella*	75	4	11	5	0	0.94
*Lactobacillus*	75	3	12	5	0	0.99
*Shuttleworthia*	75	2	13	4	1	0.28
*Tannerella*	70	9	5	4	2	0.64
*Treponema*	70	0	14	3	3	0.15
*Porphyromonas*	65	8	5	7	0	0.42
*Aggregatibacter*	65	4	9	7	0	0.79
*Abiotrophia*	65	1	12	7	0	0.75
*Peptostreptococcus*	65	1	12	7	0	0.88
*Dialister*	55	6	5	8	1	0.90
*Selenomonas*	55	6	5	8	1	0.23
*Cardiobacterium*	55	3	8	8	1	0.27
*Megasphaera*	50	8	2	9	1	0.70
*Eubacterium*	50	6	4	6	4	0.82
*Solobacterium*	50	3	7	0	10	0.59
*Actinomyces*	45	8	1	11	0	0.78
*Filifactor*	35	5	2	13	0	0.48
*Slackia*	25	4	1	0	15	0.08
*Atopobium*	5	1	0	19	0	0.22

a calculated by numbers of concordant counts/discordant counts.

b Pearson correlation based on relative intensity of HOMIM and rations of RDP classified sequence read for each genus.

## Discussion

We found that community profiles assessed by 16S rRNA pyrosequencing and HOMIM were highly correlated at the phylum level and, when comparing the more commonly detected taxa, also at the genus level. However, concordance of less common genera was weaker. While the number of genera detected in 16S rRNA pyrosequencing was greater than with HOMIM, the relative contribution of these additionally detected genera was minor, consistent with the fundamental design of the two assays: the 16S rRNA pyrosequencing assay is designed to detect broad-ranged microbiome profiles, while the custom-designed HOMIM was developed to specifically capture the major oral microbiome species.

Despite the fundamental technological differences in these approaches, it was possible to correlate number of reads in the 16S rRNA pyrosequencing data with probe intensity levels in HOMIM at the phylum and genus levels. However, due to one-to-many and many-to-one relationships between the two grouping schemes, it was not possible to accurately compare genus-level assignments. Nonetheless, we found that community profiles assessed by 16S rRNA pyrosequencing and HOMIM were highly correlated at the phylum level, and when comparing the more commonly detected taxa, at the genus level. The overall high correlation between these two high-throughput methods suggests the relative robustness of both methods.

Microarrays detect only taxa that are covered by the reference sequences. As expected, we detected greater numbers of genera with 16S rRNA pyrosequencing, compared to HOMIM, which uses pre-constructed probes designed to detect the most common bacteria in the oral cavity. In contrast, broad based 16S rRNA sequencing was able to comprehensively detect a wider range of species, particularly in rarer taxa.

At low prevalence rates, 454 pyrosequencing is more sensitive than HOMIM DNA hybridization (Unpublished data, Dr. Paster). Moderate correlations in rarer taxa could also be due to the different quantitative estimation methods: HOMIM was based on discrete numbers in an intensity scale and 16S rRNA sequencing was based on sequence reads on a continuous scale. Furthermore, laboratory assay error (i.e., cross-hybridization between probes in HOMIM and annealing bias, cloning bias and RDP misclassification [9] in 16S rRNA sequencing) could also have contributed to the discrepancies. Our findings of high correlation at the phylum level and for common genera, with relatively lower correlations for the overall genera level, are consistent with two smaller studies reporting quantitative comparisons of gut microbiome profiles using similar methods [10], [11].

In Table 4, we summarize the strengths and limitations of both assays, with respect to types of bacteria identifiable, quantification approach and ease of use. For microbiome discovery, the pyrosequencing approach has the distinct advantage of broader-spectrum identifications, although the costs and labor involved are currently somewhat greater. The approaches may, however, be similar in utility for epidemiologic investigations relating the oral microbiome to disease status. The great majority of genera are identified by both methods and epidemiologic investigations, unless very large, may not be powered to adequately investigate risk differentials related to the rarer taxa uniquely identified by pyrosequencing. For the same reasons, the capacity to find sequence reads for a large variety of rarer unclassified taxa may be of little importance at least in the earlier stages of epidemiologic investigations. Currently, these approaches provide a similar level of information for identifying etiologic associations in epidemiologic studies. Pyrosequencing does, however, provide a broader spectrum of taxa identification, has a distinct sequence-read record, may have greater detection sensitivity and, as costs and analytic complexity decrease, will likely prove in the near-term to be the method of choice for high-throughput epidemiologic investigations of the oral microbiome, at least until capacity develops for cost-effective metagenomic analysis of entire genomic ecosystems.

**Table 4:** Strengths and limitations of 16S rRNA gene pyrosequencing and Human Oral Microbe Identification Microarray (HOMIM) assay.

Pyrosequencing	HOMIM
Broad range detection of taxa	Focused detection of common known species
Detection of unclassified microbes	Custom array based approach, covered by reference sequences
Quantification based on sequence reads	Quantification based on relative intensity score
Relatively high assay cost	Relatively low assay cost
Relatively more labor intensive	Relatively less labor intensive

In summary, we found that community profiles assessed by 16S rRNA pyrosequencing and HOMIM were highly correlated at the phylum level and for the more common taxa at the genus level and we consider both methods currently suitable for high-throughput epidemiologic investigations relating the oral microbiome to disease risk; yet, pyrosequencing may provide a broader spectrum of taxa identification, a distinct sequence-read record, and greater detection sensitivity.

## Materials and Methods

### Study population

20 subjects, ages 19–89 (35% male, 65% female), were recruited at *Memorial Sloan*-Kettering *Cancer* Center, NY, in 2009. Information on basic demographic and clinical factors was obtained based on medical chart abstraction by clinicians (IG and LM). Five patients had oral cancer, 5 had premalignant oral lesions, and 10 were healthy controls. The study was approved by the institutional review boards at the Memorial Sloan-Kettering Cancer Center and NYU School of Medicine, and all participants provided informed written consent.

### Biospecimen collection and DNA extraction

Oral wash saliva samples were obtained using saline from each subject, and immediately centrifuged to harvest cell pellets. DNA was extracted from the cell pellets using the QIAamp® DNA Mini Kit (Qiagen, GmbH, Hilden, Germany) according to the instructions of the manufacturer. The extracts were stored at −20°C until use.

### 16S rRNA 454 Pyrosequencing

Bacterial 16S rRNA gene amplification, cloning, and sequencing of the polymerase chain reaction (PCR) products were performed as previously described [12], at the laboratory of Drs. Yang and Pei. 16S rRNA genes were amplified using 347F/803R primers, multiplexed with 10-mer nucleotide barcodes, and sequenced using 454 technology, that we recently designed for use in study of the foregut microbiome, targeting V3–V5 hypervariable regions and covering a sequence distance of ∼450 bp, showing close to maximum percent accuracy at this amplicon size [6].

16S rRNA sequence data from pyrosequencing was downloaded, and multiplexed samples were deconvoluted computationally using customized Perl scripts, based on the presence of the unique barcodes assigned to each sample. Initial processing steps included trimming off the barcodes and primers, and removing sequences of low quality (<Q20). The sequence reads were binned to phyla and genera using the Classifier at RDP-II [13]. For classification at the phylum to genus level, FASTA files were uploaded onto the RDPII Classifier at 80% confidence threshold and results were viewed at a display depth of 7 for assignment of data down to the genus level. The community structure of a sample was calculated based on the membership and relative abundance, based on proportion of reads, of taxonomic groups in the sample.

### HOMIM Assay

HOMIM hybridization assay [14] was conducted in duplicate at the laboratory of Dr. Paster, with previously reported protocol [3], [8]. Briefly, 16S rRNA-based, reverse-capture oligonucleotide probes (typically 18 to 20 bases) were printed on aldehyde-coated glass slides. Subject sample 16S rRNA genes were PCR amplified from DNA extracts using 16S rRNA universal forward and reverse primers and labeled via incorporation of Cy3-dCTP in a second nested PCR. The labeled 16S amplicons were hybridized overnight to probes on the slides. After washing, the microarray slides were scanned using an Axon 4000B scanner and crude data was extracted using GenePix Pro software. After microarray scanning the slides, the median background intensity for each individual feature was subtracted from the median feature intensity, yielding a normalized “median intensity score.” The generated files were exported to the HOMIM tool website (http://bioinformatics.forsyth.org/homim/) to determine the presence or absence of a particular microorganism, based on specific criteria set for that individual spot, thus generating microbial profile maps for each sample.

HOMIM output data were merged to the Human Oral Microbiome Database taxon table [7]. For each sample, we derived an estimate of the relative distribution of each taxonomic group in our phylogenetic tree using an algorithm that ensures that no species contributes more than once to the estimate of taxonomic group abundance, and that the downstream probes (probes that represent distinct subsets of species belonging to that phylogenetic group) are incorporated into the cumulative group abundance estimate. Specifically, for each phylogenetic group in each sample, all of the downstream probes were sorted according to their microarray-based relative abundance estimates to calculate the sum relative abundance for all nonoverlapping probes. As a result, the specific probes added together to represent a given taxonomic group, depending on which specific probes had the greatest hybridization signal in that sample. Spot intensities of HOMIM data were then summarized for all taxa at the phylum and genus level for each sample.

### Comparing Pyrosequencing and HOMIM Assays

Ratios of total sample intensity, from HOMIM, were then compared with corresponding ratios of numbers of RDP-classified sequence reads, from pyrosequencing, for the same sample and taxa, making comparisons at the phylum and genera levels. The relative abundance of a specific taxonomic group was compared for the two assay methods by Chi-square test. Pearson coefficients were calculated as a measure of linear correlation between sequence and intensity ratios.

## Supporting Information

Table S1 **Sequences recovered by 454 pyrosequencing for each subject samples.**
(DOC)

Table S2 **Genera detected by HOMIM and pyrosequencing.**
(DOC)

## References

[1] Peterson J, Garges S, Giovanni M, McInnes P, Wang L (2009). The NIH Human Microbiome Project.. Genome Res.

[2] Abiko Y, Sato T, Mayanagi G, Takahashi N (2010). Profiling of subgingival plaque biofilm microflora from periodontally healthy subjects and from subjects with periodontitis using quantitative real-time PCR.. J Periodontal Res.

[3] Preza D, Olsen I, Willumsen T, Boches SK, Cotton SL (2009). Microarray analysis of the microflora of root caries in elderly.. Eur J Clin Microbiol Infect Dis.

[4] Meurman J (2010). Oral microbiota and cancer.. Journal of Oral Microbiology.

[5] Buchen L (2010). Microbiology: The new germ theory.. Nature.

[6] Nossa CW, Oberdor WE, Yang L, Aas JA, Paster BJ (2010). In silico design of 16S rRNA gene primers for analysis of human foregut microbiome using next generation sequencing technology.. World Journal of Gastroenterology.

[7] Chen T, Yu WH, Izard J, Baranova OV, Lakshmanan A The Human Oral Microbiome Database: a web accessible resource for investigating oral microbe taxonomic and genomic information.. Database (Oxford).

[8] Colombo AP, Boches SK, Cotton SL, Goodson JM, Kent R (2009). Comparisons of subgingival microbial profiles of refractory periodontitis, severe periodontitis, and periodontal health using the human oral microbe identification microarray.. J Periodontol.

[9] Wang Q, Garrity GM, Tiedje JM, Cole JR (2007). Naive Bayesian classifier for rapid assignment of rRNA sequences into the new bacterial taxonomy.. Appl Environ Microbiol.

[10] Claesson MJ, O'Sullivan O, Wang Q, Nikkila J, Marchesi JR (2009). Comparative analysis of pyrosequencing and a phylogenetic microarray for exploring microbial community structures in the human distal intestine.. PLoS One.

[11] Palmer C, Bik EM, DiGiulio DB, Relman DA, Brown PO (2007). Development of the human infant intestinal microbiota.. PLoS Biol.

[12] Pei Z, Bini EJ, Yang L, Zhou M, Francois F (2004). Bacterial biota in the human distal esophagus.. Proc Natl Acad Sci U S A.

[13] Cole JR, Chai B, Farris RJ, Wang Q, Kulam SA (2005). The Ribosomal Database Project (RDP-II): sequences and tools for high-throughput rRNA analysis.. Nucleic Acids Res.

[14] Paster BJ, Dewhirst FE (2009). Molecular microbial diagnosis.. Periodontol 2000.

