# Supplementary material for: Oral Microbiome Profiles: 16S rRNA Pyrosequencing and Microarray Assay Comparison
Source: PLoS One. 2011 Jul 29;6(7):e22788. doi: 10.1371/journal.pone.0022788 (PMC3146496; doi:10.1371/journal.pone.0022788)
Supplement: Table S1 — Sequences recovered by 454 pyrosequencing for each subject samples. (DOC) [file pone.0022788.s001.doc]

Table S1. Sequences recovered by 454 pyrosequencing for each subject samples

| Subject ID | Sequences recovered by 454 pyrosequencing |
| --- | --- |
| 1 | 3211 |
| 2 | 3403 |
| 3 | 1478 |
| 4 | 2470 |
| 5 | 2154 |
| 6 | 1545 |
| 7 | 2000 |
| 8 | 2206 |
| 9 | 2393 |
| 10 | 1369 |
| 11 | 2238 |
| 12 | 1932 |
| 13 | 3042 |
| 14 | 2399 |
| 15 | 2768 |
| 16 | 2485 |
| 17 | 2897 |
| 18 | 9598 |
| 19 | 10584 |
| 20 | 18874 |
| total | 79046 |
