# Supplementary material for: Oral Microbiome Profiles: 16S rRNA Pyrosequencing and Microarray Assay Comparison
Source: PLoS One. 2011 Jul 29;6(7):e22788. doi: 10.1371/journal.pone.0022788 (PMC3146496; doi:10.1371/journal.pone.0022788)
Supplement: Table S2 — Genera detected by HOMIM and pyrosequencing. (DOC) [file pone.0022788.s002.doc]

Table S2. Genera detected by HOMIM and pyrosequencing.

| HOMIM | 454 pyrosequensing |
| --- | --- |
| **Abiotrophia** | **Abiotrophia** |
| **Actinomyces** | Acholeplasma |
| **Aggregatibacter** | Actinobacillus |
| **Atopobium** | **Actinomyces** |
| Bacteroides | **Aggregatibacter** |
| Bacteroidetes | Alloscardovia |
| Bergeyella | Anaerococcus |
| Bifidobacteriaceae | Anaeroglobus |
| **Bifidobacterium** | Arcanobacterium |
| **Campylobacter** | **Atopobium** |
| **Capnocytophaga** | Bergeriella |
| **Cardiobacterium** | **Bifidobacterium** |
| **Catonella** | Butyrivibrio |
| Clostridiales | **Campylobacter** |
| **Dialister** | **Capnocytophaga** |
| Eikenella | **Cardiobacterium** |
| Enterococcus | **Catonella** |
| **Eubacterium** | Centipeda |
| **Filifactor** | Conchiformibius |
| **Fusobacterium** | Corynebacterium |
| **Gemella** | Cryptobacterium |
| **Granulicatella** | **Dialister** |
| **Haemophilus** | **Eubacterium** |
| **Kingella** | **Filifactor** |
| Lachnospiraceae | **Fusobacterium** |
| **Lactobacillus** | **Gemella** |
| **Lactococcus** | **Granulicatella** |
| Lautropia | **Haemophilus** |
| **Leptotrichia** | Hallella |
| **Megasphaera** | Helcococcus |
| **Mycoplasma** | Howardella |
| **Neisseria** | Johnsonella |
| **Parvimonas** | **Kingella** |
| **Peptostreptococcus** | **Lactobacillus** |
| **Porphyromonas** | **Lactococcus** |
| **Prevotella** | Lactovum |
| Propionibacterium | **Leptotrichia** |
| **Rothia** | Mannheimia |
| **Scardovia** | **Megasphaera** |
| **Selenomonas** | Mobiluncus |
| **Shuttleworthia** | Mogibacterium |
| **Slackia** | Moraxella |
| **Solobacterium** | Moryella |
| Sphaerocytophaga | **Mycoplasma** |
| Stenotrophomonas | **Neisseria** |
| **Streptococcus** | Nicoletella |
| **Tannerella** | Olsenella |
| TM7 | Oribacterium |
| **Treponema** | Paraprevotella |
| **Veillonella** | Parascardovia |
|  | **Parvimonas** |
|  | Pasteurella |
|  | Peptococcus |
|  | Peptoniphilus |
|  | **Peptostreptococcus** |
|  | Phocaeicola |
|  | Planobacterium |
|  | **Porphyromonas** |
|  | **Prevotella** |
|  | Pseudoramibacter |
|  | Pyramidobacter |
|  | **Rothia** |
|  | **Scardovia** |
|  | **Selenomonas** |
|  | Serratia |
|  | **Shuttleworthia** |
|  | **Slackia** |
|  | **Solobacterium** |
|  | SR1_genera |
|  | Streptobacillus |
|  | **Streptococcus** |
|  | Streptophyta |
|  | Syntrophococcus |
|  | **Tannerella** |
|  | TM7 |
|  | **Treponema** |
|  | Vagococcus |
|  | **Veillonella** |

Note: Bold: common genera detected by both HOMIM and pyrosequencing.
